# Psychotic Disorders in Adolescence and Later Long-term Exclusion From Education and Employment

**DOI:** 10.1093/schbul/sbac151

**Published:** 2022-10-28

**Authors:** Ida Ringbom, Jaana Suvisaari, Antti Kääriälä, Andre Sourander, Mika Gissler, Ian Kelleher, David Gyllenberg

**Affiliations:** Department of Child Psychiatry and Invest Flagship, University of Turku, Finland; Finnish Institute for Health and Welfare, Finland; Helsinki University Hospital, Department of Adolescent Psychiatry, Finland; Finnish Institute for Health and Welfare, Finland; Finnish Institute for Health and Welfare, Finland; Department of Child Psychiatry and Invest Flagship, University of Turku, Finland; Turku University Central Hospital, Department of Child Psychiatry, Finland; Department of Child Psychiatry and Invest Flagship, University of Turku, Finland; Finnish Institute for Health and Welfare, Finland; Karolinska Institute, Department of Molecular Medicine and Surgery, Sweden and Region Stockholm, Academic Primary Health Care Centre, Stockholm, Sweden; School of Medicine, University College Dublin, Ireland; Department of Child Psychiatry and Invest Flagship, University of Turku, Finland; Finnish Institute for Health and Welfare, Finland; Helsinki University Hospital, Department of Adolescent Psychiatry, Finland

**Keywords:** functional disability, labor market, rehabilitation, NEET, schizophrenia

## Abstract

**Background and hypothesis:**

Psychotic disorders have been associated with not being in education, employment, and training (NEET). There is a lack of knowledge on the importance of risk markers for NEET among people with psychotic disorders and what rehabilitation they receive.

**Study design:**

We based our research on the register-based 1987 Finnish Birth Cohort study, which included all live births in Finland during that year. The study cohort were 288 people who had been diagnosed with psychotic disorders during 2004–2007, when they were 16–20 year old, and 55 883 who had not. We looked at the national register data for those subjects in 2008–2015, when they were 20–28 year old, and compared any associations between sociodemographic factors and NEET status.

**Study results:**

NEET for more than 5 year affected 2.2% of those without psychosis, 35.8% of those with any nonaffective psychotic disorder, and 57.0% of those with schizophrenia or schizoaffective disorders. Family-related risk factors were weaker predictors of long-term NEET in subjects with psychotic disorders than other cohort members. Having a psychotic disorder plus long-term NEET was associated with not applying for upper secondary education, not finishing upper secondary education, parents receiving welfare benefits, being diagnosed with schizophrenia or schizoaffective disorders and being hospitalized for psychosis. Only 24.3% with psychotic disorders had participated in vocational rehabilitation.

**Conclusions:**

A diagnosis of psychosis in adolescence is independently associated with serious long term functional disability. Among those with psychotic disorders, educational problems are markers for adverse labor market outcomes. Despite this, vocational rehabilitation is seldom provided.

## Introduction

Work plays a critical role in the lives and recoveries of people with psychotic disorders. A job provides financial independence, structure, purpose, relationships, self-worth, meaning in life, and a social role without stigma.^1^ Psychiatric disorders, in particular, nonaffective psychosis, continue to be associated with spending time Not in Education, Employment, or Training (NEET) in western countries.^2,3^

Epidemiological studies have shown that many people with psychotic disorders are outside of the labor market^3–7^ and interventions that have aimed to help people enter, or return to, work have had poor participation rates.^8^ Educational problems have been associated with psychosis, according to the neurodevelopmental model,^9^ and with labor market marginalization.^10^ Socioeconomic disadvantages have also been associated with labor market marginalization^10^ and with psychosis by some models.^11^ It is important to establish the separate associations between educational problems, socioeconomic disadvantages, psychosis, and labor market marginalization and how these relate to participation in rehabilitation programmes. To our knowledge, no studies to date have compared risk markers for labor market marginalization in the general population and people who have been diagnosed with psychotic disorders.

Our first aim was to assess labor market outcomes for people diagnosed with psychotic disorders in adolescence and our main outcome of interest was people who had been long-term NEET for more than five years. The second aim was to investigate risk markers for long-term NEET among people who had been diagnosed with a psychotic disorder in adolescence and the general population. The third aim was to examine whether socioeconomic and educational factors, which have both been associated with NEET and psychosis, would also be associated with using rehabilitation services.

## Methods

### Study Design

We used data from the 1987 Finnish Birth Cohort study, which has previously been described in detail.^12^ That longitudinal study is managed by the Finnish Institute for Health and Welfare and contains data from Finnish nationwide registers for all children born in Finland in that year. The study was approved by the Institute’s review board, and we obtained permission from the registered keepers to use the data sources, as required by Finnish law. All data were pseudonymised and handled according to Finnish data protection legislation and regulations. Informed consent was not required, because none of the individuals who were included were contacted.

**Fig. 1.**
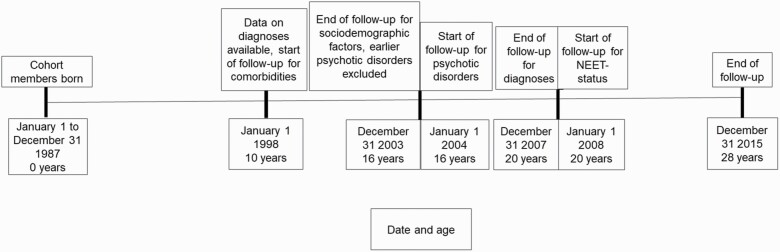
Timeline showing the main phases of the study.

The population-based cohort was comprised of 59 476 people who were born in Finland in 1987. We excluded individuals who had lived outside Finland or died before the end of 2015, had a diagnosis of intellectual disability or had been diagnosed with a psychotic disorder from 1998 to 2003. We chose a start date of 1998, because the relevant registers did not provide outpatient data before that year. The end date of 2003 enabled us to follow up sociodemographic factors in individuals until completion of compulsory education, at the age of 15–16 year, before their first diagnosis of psychosis. As problems in education have been associated with long-term NEET status in young people with psychotic disorders,^2^ we wanted to see if these kinds of problems had been present already before the diagnosis. See figure 1 for a timeline.

### Description of *the* Registers

Data from different sources were linked to each individual using the unique personal identification code that is assigned to Finnish citizens and permanent residents. Data from nine registers were used for this study. Statistics Finland provided the education levels for the parents of the cohort members and when any deceased cohort members died. The Medical Birth Register identified the subjects’ mothers. The Digital and Population Data Services Agency provided emigration data and identified the subjects’ fathers. Data on welfare benefits was provided by The Finnish Institute for Health and Welfare and salaries and childcare benefits by the Finnish Centre for Pensions. The Social Insurance Institution provided data on financial support for rehabilitation, student benefits, and purchase of medications. The Ministry of Economic Affairs and Employment register provided information about whether the subjects had participated in jobseekers’ programmes. The Joint Application Register provided information about school grades. These registers have previously been described in detail.^13,14^

Information on diagnoses were obtained from the Finnish Care Register for Health Care. These were recorded during visits to physicians at inpatient or outpatient clinics in hospitals and psychiatric specialized health care services across Finland. The records are regularly submitted to the Register by the hospital districts. The Register includes the start and end dates of the visits, a mandatory primary diagnosis and optional secondary diagnoses and has contained inpatient data since 1967 and outpatient data since 1998. This register has been widely used for epidemiological research^14^ and the diagnostic validity of diagnoses of schizophrenia in the Register has been confirmed.^15^

### Definitions of Outcomes

The main outcome was long-term NEET when the cohort members were 20–28 year old.^2^ Subjects were regarded as long-term NEET if they had not been working, studying on parental leave or taking part in jobseekers’ programmes for five calendar years between 2008 and 2015. Employment was defined as receiving any salary that contributed to a pension scheme. Being in education was defined as receiving any student benefits. Parental leave was defined as receiving childcare benefits. Participating in jobseekers’ programmes was counted as training. We also examined the different occupational statuses separately. We chose to study long-term NEET, rather than being NEET for a short time such as one week or even a year, because it is a more severe marker of labor market marginalization.^2^

### Definition of Service Use for Psychotic Disorders

Psychosis was defined as a diagnosis of nonaffective psychosis between 2004 and 2007 when the cohort members were 16–20 year old. The diagnoses were recorded according to International Statistical Classification of Diseases and Related Health Problems, Tenth Edition (ICD-10) codes of F20–F29. Primary and secondary diagnoses recorded by inpatient or outpatient services were included.

If an individual had been diagnosed with a psychotic disorder, we looked for whether they had specific clinical characteristics that have been associated with adverse labor market outcomes.^5^ These were a diagnosis of schizophrenia or schizoaffective disorder, whether they had been hospitalized for a psychotic disorder and whether the first diagnosis of a psychotic disorder had been made by the year 2005, when the cohort members turned 18.

### Socio-economic Background Factors and Comorbidities

The potential risk markers for labor market marginalization were derived from the registers and we selected background factors that have previously been associated with adverse labor market outcomes.^1,3–7,10,16–18^ We considered four socioeconomic factors related to the parents: educational attainment, marital status, whether they had received any welfare benefits, and whether they had been hospitalized with a mental health disorder. Parental education was divided into three categories, based on the highest educational attainment by either parent: compulsory education (9 year of education), upper secondary education (12 year), or further education (≥13 year). The combined model used two categories: compulsory education and any education after those first 9 year. Parents were defined as married if they were married for the whole of 1987–2003. They were defined as not married if they had never been married or they had separated or one of the parents had died before 2004.

We looked at two background factors related to the subjects’ educational attainment: their school grades and whether they had achieved an upper secondary education diploma by the end of the year 2008, when they were at age 21. The register only included grade data for subjects who had applied for upper secondary education through the national application system. School grades were assessed at the end of spring 2003, as this is when most subjects would have completed their compulsory education. If their 2003 grades were missing, we counted grades from 2004 to allow for subjects who had started or finished their education later than expected. The school records included their average grade for all the compulsory subjects. We divided the average grades into three categories, based on whether the grades were average or higher than average, below average or missing. These two factors were studied separately and combined with diagnoses of any learning disability (ICD-10 F80–83). In the combined model, low and missing grades provided a combined risk marker.

The last socioeconomic risk marker we studied was being placed outside the home by the child protection agency before 2004.

We looked for a number of ICD-10 psychiatric diagnosis codes during 1998–2007 that indicated possible comorbidities of psychotic disorders that have been found to be risk factors for long-term NEET.^2^ These were: substance use disorders (F10-–F19), depressive disorders (F32–F33), anxiety disorders (F40–F48), eating disorders (F50), learning disabilities (F80–83), autism spectrum disorders (F84), and conduct disorders (F90.1, F91). We did not include diagnoses before 1998, as no outpatient data were recorded before that year.

### Rehabilitation

We looked at whether subjects had received financial support from the Social Insurance Institution of Finland for rehabilitation to increase their employability.^19^ Rehabilitation was categorized into vocational rehabilitation, psychotherapy, and other. We studied people diagnosed with a psychotic disorder to see if there were any associations between parental risk markers or risk markers related to learning and education and engaging in vocational rehabilitation. These were separately analyzed for those who had a psychotic disorder and were, or were not, long-term NEET.

### Statistical Methods

We started by comparing the proportions of different labor market outcomes and risk markers, stratified by psychosis status.

Due to the binary outcomes, we used logistic regression to carry out statistical modeling for the association between psychotic disorders and long-term NEET. We studied one predictor at a time in univariate models. We calculated the various interactions separately to compare the associations between the sociodemographic characteristics and long-term NEET among those with, and without, psychotic disorders.

R statistical software, version 3.4.0 (R Foundation, Vienna, Austria) was used for the analyses.

## Results

### Participants and Setting

The 1987 Finnish Birth Cohort study included 59 476 people who were born in Finland in that year and survived the perinatal period. Of these, 756 had died before the end of follow-up period in 2015, 2 913 had emigrated, and 534 had been diagnosed with intellectual disabilities. A further 117 were excluded because they were diagnosed with a psychotic disorder during 1998–2003, before the start of the follow-up period. The final number included in the analyses was 55 171, which was 92.8% of the cohort. Of these 288 (0.5%) had been diagnosed with any nonaffective psychotic disorder during 2004–2007 and these included 68 with schizophrenia (ICD-10-code F20), 18 with schizoaffective disorder (F25), 50 with acute and transient psychotic disorders (F23), and 34 with other specified types of nonaffective psychosis (F21–22, F24, and F28). The rest, 118 cohort members, had only a diagnosis of unspecified psychosis (F29).

### Labor Market Outcomes

The labor market outcomes are presented in table 1. Studying, working, and being on parental leave was less common among those with psychotic disorders than those without. The 86 subjects diagnosed with schizophrenia or schizoaffective disorders were more likely to be not studying (*n* = 61, 70.9%), not working (*n* = 40, 46.5%), or not on parental leave (*n* = 75, 87.2%) than the 288 with any psychosis, including those two diagnoses, or the 54 883 with no psychosis at all.

**Table 1.** Status during 2008–2015 when the subjects were 21–28 years of age

Characteristic	Time span	No psychosis (*n* = 54 883)	Any psychosis (*n* = 288)	Schizophrenia (n = 86)1
Studying	0 year	18 210 (33.2%)	159 (55.2%)	61 (70.9%)
	1–4 year	16 515 (30.1%)	84 (29.2%)	18 (20.9%)
	≥ 5 year	20 158 (36.7%)	45 (15.6%)	7 (8.1%)
Working	0 year	1430 (2.6%)	85 (29.5%)	40 (46.5%)
	1–4 year	5469 (10.0%)	101 (35.1%)	29 (33.7%)
	≥ 5 year	47 984 (87.4%)	102 (35.4%)	17 (19.8%)
Parental leave	0 year	39 388 (71.8%)	233 (80.9%)	75 (87.2%)
	1–4 year	14 503 (26.4%)	50 (17.3%)	11 (12.8%)
	≥ 5 year	1042 (1.9%)	5 (1.7%)	0 (0.0%)
Training	Any time	19 861 (36.2%)	138 (47.9%)	31 (36.0%)
Not in education, employment or training	0 year	45875 (83.6%)	99 (34.4%)	13 (15.1%)
	1 4 year	7821 (14.3%)	86 (29.9%)	24 (27.9%)
	≥ 5 year	1187 (2.2%)	103 (35.8%)	49 (57.0%)

^1^Schizophrenia cases were a subgroup of psychosis cases and also included schizoaffective disorders.

The 288 subjects with psychotic disorders were more likely to have taken part in training provided by the unemployment agency (47.9%) than those without a psychotic disorder (36.2%). The percentage for the subgroup with a diagnosis of schizophrenia or schizoaffective disorder was 36.0%.

Nearly two-thirds (65.6%) of those with psychotic disorders had been NEET for at least a year and 35.8% had been long-term NEET for at least 5 year. The corresponding figures for those with schizophrenia or a schizoaffective disorder were 84.9% and 57.0%, respectively. These figures were significantly higher than the 16.4% and 2.2% for those subjects with no psychoses, respectively.

The odds ratio for being long-term NEET was 23.1, with a 95% confidence interval (CI) of 18.0–29.5 (*P* < .001), for those with psychotic disorders compared to the full cohort.

### Sociodemographic Characteristics and Comorbidities

The sociodemographic characteristics of the sample are shown in table 2. All potential risk markers for long-term NEET, except for male sex, were more common in the group with psychotic disorders than the rest of the cohort. More than half (58.3%) of those with psychotic disorders had a parent who had received welfare benefits. We found that 12.6% of those with psychotic disorders who had been long-term NEET had not applied for upper secondary education when they finished their compulsory education and 78.6% did not have an upper secondary education diploma by the end of 2008.

**Table 2.** The prevalence of sociodemographic characteristics and long-term NEET among those with and without a psychotic disorder.

	No psychosis diagnosed			Psychosis diagnosed		
	Total (*n* = 54 883)	NEET (*n* = 1290)	Proportion who are NEET	Total (*n* = 288)	NEET (*n* = 103)	Proportion who are NEET
**Risk marker**	N (%)	N (%)	%	N (%)	N (%)	%
*Parental markers*						
Severe psychiatric illness	5847 (10.7)	244 (18.9)	4.2	75 (26.0)	34 (33.0)	45.3
Not married	27385 (49.9)	828 (64.2)	3.0	173 (60.1)	59 (57.3)	34.1
Welfare support	19964 (36.4)	698 (54.1)	3.5	143 (49.7)	60 (58.3)	42.0
Education Compulsory	4086 (7.4)	170 (13.2)	4.2	33 (11.4)	15 (14.6)	45.5
Upper secondary	23974 (43.7)	639 (49.5)	2.7	114 (39.6)	41 (39.8)	36.0
Further education	26823 (48.9)	481 (37.3)	1.8	141 (49.0)	47 (45.6)	33.3
At least one parental marker	33967 (61.9)	981 (76.0)	2.9	208 (72.2)	81 (78.6)	38.9
*Learning and educational markers*						
No upper secondary education	9106 (16.6)	815 (63.2)	9.0	170 (59.0)	81 (78.6)	47.6
School grades¹ Average or higher	49109 (89.5)	813 (63.0)	1.7	235 (81.6)	77 (74.8)	32.8
Low	3925 (7.2)	210 (16.3)	5.4	30 (10.4)	13 (12.6)	43.3
Missing	1849 (3.4)	267 (20.7)	14.4	23 (8.0)	13 (12.6)	56.5
Learning disability	792 (1.4)	84 (6.5)	10.6	15 (5.2)	9 (8.7)	60.0
At least one learning or educational marker	12415 (22.6)	893 (69.2)	7.2	179 (62.2)	83 (80.6)	46.3
*Other markers*						
Child protection	1455 (2.7)	163 (12.6)	11.2	35 (12.2)	15 (14.6)	42.9
Male	28281 (51.5)	860 (66.7)	3.0	135 (46.9)	54 (52.4)	40.0
Substance use disorders	724 (1.3)	93 (7.2)	12.8	61 (21.2)	21 (20.4)	34.4
Depressive disorders	2167 (3.9)	211 (16.4)	9.7	152 (52.8)	59 (57.3)	38.8
Anxiety disorders	1866 (3.4)	185 (14.3)	9.9	95 (33.0)	36 (35.0)	37.9
Eating disorders	496 (0.9)	26 (2.0)	5.2	14 (4.9)	6 (5.8)	42.9
Autism spectrum disorders	105 (0.2)	45 (3.5)	42.9	12 (4.2)	5 (4.9)	41.7
Conduct disorders	695 (1.2)	97 (7.5)	14.0	30 (10.4)	12 (11.7)	40.0

^¹^Average or higher = average grade above -1.5 SD from the average, Low = average grade below -1.5 SD from the average, Missing = no registered average school grade.

Subjects diagnosed with a psychotic disorder had the following comorbidities: substance use disorder (20.4%), depressive disorder (57.3%), learning disabilities (8.7%), autism spectrum disorder (4.9%), and conduct disorder (11.7%). We found no significant association between those comorbidities and being long-term NEET. Despite this, those with both a psychotic disorder and a learning disability were most likely to be long-term NEET (60.0%).

**Fig. 2.**
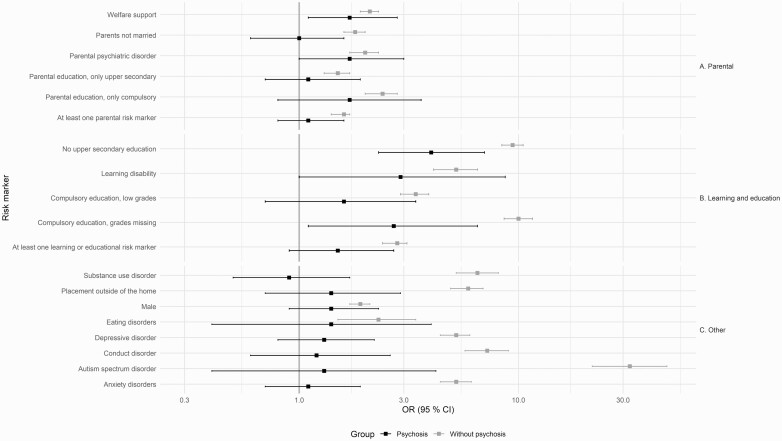
Sociodemographic characteristics related to long-term NEET-status in young adulthood, with separate univariate analyses for those with and without psychotic disorders. The odds ratios (OR) and 95% confidence intervals (CI) are presented on a logarithmic scale.

The univariate results for the associations between all of these diagnoses and long-term NEET are presented in figure 2. There was a statistically significant association between long-term NEET and all the sociodemographic factors and the psychiatric comorbidities we studied (*P* < .001).

The odds ratio for having psychotic disorders and being long-term NEET was 2.7 (95% CI 1.1.–6.5, *P* < .05) for those who did not apply for upper secondary education, 4.0 (95% CI 2.3–7.0, *P* < .001) for those who did not have an upper secondary education diploma and 1.7 (95% CI 1.1–2.8, *P* < .05) for parents on welfare benefits. There were no significant associations with the other sociodemographic characteristics in subjects with psychotic disorders who were long-term NEET.

There was an interaction with *P* < .05 between psychotic disorders and being placed in care outside the home (*P* < .001), missing school grades from compulsory education (*P* = 0.0032), not having a diploma from upper secondary education (*P* = 0.0029), parents not being married (*P* = 0.01) and all studied comorbidities except for eating disorders and learning disabilities. However, when we applied the Bonferroni correction only placement outside of the home, not having a diploma from upper secondary education and the comorbidities were significant. The association between these factors and being long-term NEET were significantly weaker for those with a psychotic disorder than for the full cohort. (Supplemental Table 1)

### Clinical Characteristics

We carried out three separate univariate analyses to characterize the potential clinical risk factors for long-term NEET among the 288 subjects diagnosed with psychotic disorders. These were for the 137 (47.6%) with schizophrenia or a schizoaffective disorder, the 123 (42.7%) diagnosed by 2005 at the latest, when the cohort members turned 18, and the 178 (61.8%) treated as inpatients (Supplementary Table 1).

The odds ratio for long-term NEET was 3.6 (95% CI 2.1–6.2, *P* < .001) for those with schizophrenia or schizoaffective disorder vs any other diagnosis of nonaffective psychotic disorder. The odds ratio was 2.9 (95% CI 1.7-5.0, *P* < .001) for those who had been inpatients vs those who had not been. There was no statistical difference between those diagnosed 2004–2005 (age 16–18) vs those diagnosed during 2006–2007 (age 18–20).

We also made additional analysis on what kind of treatment the cohort members had received during the years 2004–2007. Of those diagnosed with a psychotic disorder 229 (79.5%) had bought an antipsychotic from a pharmacy. Most (74.3%) had visited a doctor at an outpatient clinic in specialized health care, 58 (20.1%) had one visit recorded, 45 (15.6%) had 2–5 visits, 33 (11.5%) had 6–10 visits, 78 (27.1%) had more than ten visits.

### Rehabilitation

Only 36.8% of those diagnosed with a psychotic disorder had received rehabilitation funded by The Social Insurance Institution of Finland. Vocational rehabilitation was the most common form, and this was provided in 70 (24.3%) of cases. Vocational rehabilitation was more common among those who were long-term NEET than those who were not (34.0% vs 18.9%). Only 8.0% received rehabilitative psychotherapy and 11.8% received other types of rehabilitation.

As vocational rehabilitation is specifically aimed at securing employment, we examined the associations between risk markers and vocational rehabilitation among those with a psychotic disorder who were and were not long-term NEET. When we looked at those with a psychotic disorder who were long-term NEET, we found a negative association between having at least one adverse family characteristic and taking part in vocational rehabilitation (OR 0.3,0 95% CI 0.1–0.7, *P* < .01). There was no association between problems at school and taking part in vocational rehabilitation (table 3).

**Table 3.** Sociodemographic characteristics and rehabilitation. Prevalence and results of the univariate analyses in subjects with and without long-term NEET.

	Psychosis, no NEET ≥5 years				Psychosis, NEET ≥5 years			
	Total (*n* = 185)	Vocational rehabilitation (*n* = 35)			Total (*n* = 103)	Vocational rehabilitation (*n* = 35)		
Risk marker	N (%)	N (%)	OR (95% CI)	p	N (%)	N (%)	OR (95% CI)	p
Parental risk markers								
None	58 (31.4)	9 (25.7)	1		22 (21.4)	13 (37.1)	1	
At least one	127 (68.6)	26 (74.3)	1.4 (0.6-3.4)	0.426	81 (78.6)	22 (62.9)	0.3 (0.1-0.7)	0.007
Learning and educational risk markers								
None	89 (48.1)	19 (54.3)	1		20 (19.4)	9 (25.7)	1	
At least one	96 (51.9)	16 (45.7)	0.7 (0.3-1.5)	0.418	83 (80.6)	26 (74.3)	0.6 (0.2-1.5)	0.250

## Discussion

Long-term (more than 5 year) NEET was uncommon in the general Finnish population, affecting only 2% of individuals born in 1987. However, 36% of young people with a history of psychotic disorder were long-term NEET, rising to 57% for those diagnosed with schizophrenia or a schizoaffective disorder. This highlights serious problems with achieving functional recovery in people with psychosis, especially those with schizophrenia or a schizoaffective disorder.^20^ It is also important to note that 71% of the 288 diagnosed with a psychotic disorder by 2004–2007 had worked at some point during 2008–2015 and the figure for those with a diagnosis of schizophrenia or a schizoaffective disorder was 54%.

One possible reason for the association between psychotic disorders in adolescence and employment might be to do with when the disorder starts. Adolescence is a critical stage for development, as it is a key period for education, training and preparing to enter the workforce. The association between higher levels of education and positive work outcomes is well-known,^1^ so the onset of a severe mental illness during adolescence is likely to have more severe negative consequences for education and subsequent employment.

The associations between all the sociodemographic factors we studied and long-term NEET had lower ORs for the cohort members with a history of psychotic disorders. Most of the risk factors were more common in those with a history of psychotic disorders than in the general population. Although these associations were weaker, there were still strong negative associations between educational attainment and long-term NEET in early adulthood for people with a history of nonaffective psychotic disorders in adolescence. This was in line with previous research and underlines the importance of supporting people diagnosed with psychotic disorders to continue their education and to gain qualifications.^10,18^

Those who had been diagnosed with a psychotic disorder, had learning difficulties and had not applied for upper secondary education or received a diploma at that level were more likely to be NEET in later life. These educational problems could have been caused by impaired cognition, in line with the neurodevelopmental model of psychosis.

Only 24.3% with psychotic disorders had received specialized vocational rehabilitation to help them find employment. Nearly twice as many (47.9%) had participated in mainstream training programmes for jobseekers, even though research has shown that young people with severe mental disorders are unlikely to benefit from more basic employment programmes.^21^

Individuals with psychotic disorders were more likely to take part in vocational rehabilitation if they were long-term NEET than if they were NEET for less than five years. This might be a sign that vocational rehabilitation is used late, when the person already has been outside of employment, education, and training for several years or that it does not help people to enter the labor market. This is a problem, as we know that the longer a person spends away from school or work, the less likely they are to return to education or employment.^22^ The evidence-based individual placement and support model for vocational rehabilitation aims to get people into the labor market quickly and helps them to develop the skills they need to manage symptoms in the workplace. This model has been shown to produce significantly better outcomes for young adults than traditional rehabilitation programmes, which are based on waiting for symptoms to go into remission before considering work.^23,24^ The model is currently being piloted in Finland.^25^

We found a negative association between people who were long-term NEET and had an adverse family background and taking part in vocational rehabilitation. High-quality vocational rehabilitation programmes include practical training in work-related social skills, coping skills and problem-solving skills. Employment specialists need to be comprehensively trained and capable of building good relationships with employers and those who take part in programmes. The rehabilitation programmes need to be flexible and adaptable for varying degrees of disability and the emphasis should be on finding a job that fits each individual’s skills and experiences. Participants also need support from healthcare services, their families, and employers.^26^ More studies are needed on why so few people with psychotic disorders appear to receive vocational rehabilitation in Finland. This should include identifying and addressing any barriers to access.

### Strengths and Limitations

The main strength of the study was that it involved all people born in Finland born in 1987 and followed them up to 28 year of age. The other main strength was that we were able to link a comprehensive list of registers to the cohort. Finnish administrative registers have been shown to provide high coverage. Furthermore, the registers enabled us to assess NEET during a long follow-up period, as they did not suffer from attrition bias. The diagnostic validity of schizophrenia in the Care Register for Health Care has been shown to be good.^15^

A few limitations should be considered. First, only diagnoses of psychotic disorders made by specialist healthcare services could be analyzed, but these services deliver the vast majority of these diagnoses in Finland. Second, participation in jobseekers’ programmes were counted as training and these people were not defined as NEET. We made this choice as people who took part in these programmes were not marginalized, as they were actively engaging in society. These programmes can sometimes be similar to vocational rehabilitation. It is unlikely that excluding these people significantly changed the results. Third, while the registers contained information on several covariates related to marginalization, we lacked information about protective factors and resilience. School performance is only a crude indicator of cognitive capacity. Future research should investigate possible modifiers of the relationship between psychosis and NEET. These could include cognition and factors such as support from the person’s social network, motivation, self-efficacy, stigma, metacognition, and functional capacity skills.^1,27^ The registers do not either provide information on race or ethnicity. Finally, Finland provides universal and effective healthcare,^28^ but studies in other countries could provide further information, as differences in health and rehabilitation services, social benefits and the labor market might affect the associations.^24^ Studies in countries with an ethnically less homogenous population could also give answers about the role of ethnicity or migrant background in labor market marginalization among people with psychotic disorders.

## Conclusions

More than a third (35.8%) of individuals who experienced the onset of psychosis in adolescence met the criteria for long-term NEET, for more than 5 year, in young adulthood. Furthermore, 57.0% of individuals diagnosed with schizophrenia or schizoaffective disorders were long-term NEET, highlighting the serious long-term functional morbidity associated with such a diagnosis in adolescence. Individuals with a history of inpatient admission had particularly poor work outcomes and we, therefore, recommend that they should receive intensive rehabilitation as early as possible in the course of their illness. Despite the high prevalence of NEET in individuals diagnosed with a psychotic disorder, only a small proportion received vocational rehabilitation. The association between a number of sociodemographic factors and long-term NEET in early adulthood (figure 1) was weaker for those with psychotic disorders than in the general population, but problems in education is a marker for elevated risk of adverse labor market outcomes also among those with psychotic disorders.

## Supplementary Material

sbac151_suppl_Supplementary_Material
