# Supplementary material for: Psychotic Disorders in Adolescence and Later Long-term Exclusion From Education and Employment
Source: Schizophr Bull. 2022 Oct 28;49(1):90–8. doi: 10.1093/schbul/sbac151 (PMC9810018; doi:10.1093/schbul/sbac151)
Supplement: sbac151_suppl_Supplementary_Material [file sbac151_suppl_supplementary_material.docx]

**Supplemental Table 1. Sociodemographic characteristics related to long-term NEET-status in young adulthood, with separate univariate analyses for those with and without psychotic disorders. Interactions between psychotic disorders and sociodemographic characteristics for long-term NEET status.**

|  | No psychosis | Psychosis | Interaction |
| --- | --- | --- | --- |
|  | OR (95% CI)^1^ | OR (95% CI)^1^ | p |
| Parental psychiatric disorder | 2.0 (1.7-2.3) | 1.7 (1.0-3.0) | 0.613 |
| Parents not married | 1.8 (1.6-2.0) | 1.0 (0.6-1.6) | 0.011 |
| Welfare support | 2.1 (1.9-2.3) | 1.7 (1.1-2.8) | 0.426 |
| Parental education, only compulsory | 2.4 (2.0-2.8) | 1.7 (0.8-3.6) | 0.378 |
| Parental education, only upper secondary | 1.5 (1.3-1.7) | 1.1 (0.7-1.9) | 0.287 |
| No upper secondary education | 9.4 (8.4-10.5) | 4.0 (2.3-7.0) | 0.003^2^ |
| Compulsory education, low grades | 3.4 (2.9-3.9) | 1.6 (0.7-3.4) | 0.058 |
| Compulsory education, grades missing | 10.0 (8.6-10.5) | 2.7 (1.1-6.5) | 0.003 |
| Learning disability | 5.2 (4.1-6.5) | 2.9 (1.0-8.7) | 0.280 |
| Placement outside of the home | 5.9 (4.9-6.9) | 1.4 (0.7-2.9) | <0.001^2^ |
| Male | 1.9 (1.7-2.1) | 1.4 (0.9-2.3) | 0.238 |
| Substance use disorder | 6.5 (5.2-8.1) | 0.9 (0.5-1.7) | <0.001^2^ |
| Depressive disorder | 5.2 (4.4-6.0) | 1.3 (0.8-2.2) | <0.001^2^ |
| Anxiety disorders | 5.2 (4.4-6.1) | 1.1 (0.7-1.9) | <0.001^2^ |
| Eating disorders | 2.3 (1.5-3.4) | 1.4 (0.4-4.0) | 0.370 |
| Autism spectrum disorder | 32.2 (21.7-47.5) | 1.3 (0.4-4.2) | <0.001^2^ |
| Conduct disorder | 7.2 (5.7-9.0) | 1.2 (0.6-2.6) | <0.001^2^ |
| ^1^Abbreviations: OR = odds ratio, CI = Confidence interval  ^2^Below the alpha for Bonferroni 0.003 (missing school grades from compulsory education (p=0.0032), not having a diploma from upper secondary education (p=0.0029)) | | | |

**Supplemental Table 2.** Clinical factors and long-term NEET among people diagnosed with a psychotic disorder. Prevalence and results of the univariate analyses of long-term NEET in subjects with the characteristics compared with those without the characteristic.

|  | n | NEET (n=103) | OR (95 % CI) | p |
| --- | --- | --- | --- | --- |
| Schizophrenia or schizoaffective disorder | 86 | 49 (57.0%) | 3.6 (2.1-6.2) | <0.001 |
| Early onset | 105 | 44 (41.9%) | 1.2 (0.7-2.0) | 0.471 |
| Hospitalisation |  |  |  |  |
| At least one time | 178 | 79 (44.4%) | 2.9 (1.7-5.0) | <0.001 |
| One time | 93 | 37 (39.8%) | 2.4 (1.3-4.4) | 0.006 |
| Twice | 45 | 18 (40.0%) | 1.5 (1.1-2.3) | 0.022 |
| At least three times | 40 | 24 (60.0%) | 5.4 (2.5-11.9) | <0.001 |
